# Development of youth tennis players: A study based on the ranking history of top ATP/WTA players worldwide and China

**DOI:** 10.1371/journal.pone.0289848

**Published:** 2023-11-10

**Authors:** Hui Chen, Caifeng Li, Xianlu Meng, Paweł Chmura, Xiaobin Wei

**Affiliations:** 1 School of Sports and Health Management, Chongqing University of Education, Chongqing, China; 2 China Institute of Sport Science, Sports Training Research Center, Beijing, China; 3 Department of Team Games, Wroclaw University of Health and Sport Sciences, Wrocław, Poland; 4 School of Strength and Conditioning Training, Beijing Sport University, Beijing, China; Università degli Studi di Milano: Universita degli Studi di Milano, ITALY

## Abstract

**Background:**

The top 100 ATP/WTA ranking points are a crucial indicator of entry into the high-level world of tennis players, and the number of players from a nation in this ranking reflects the overall level of their tennis. However, the growth time series characteristics of elite tennis athletes are unclear.

**Objective:**

This study aims to examine the historical career ranking changes of elite players and provide valuable insights for aspiring young players looking to achieve success in the sport. At the same time, it is of great significance for the efficient and sustainable cultivation of Chinese tennis players.

**Methods:**

Data on the rankings of 202 players were analyzed, Spearman and Pearson correlations were employed to investigate the association between ranking and time-use patterns. The variance test was utilized to compare disparities in time characteristics of the ranking, with a statistical significance level of p<0.05.

**Results:**

There was a significant correlation between the time of entering the professional tournament ranking system and the ranking, top 100 time, top 100 age, and age of starting tennis. Top 50 ATP players are earlier than those ranked 51–100. There was a significant difference between the age of starting tennis and the time to top 10 among the ATP and WTA players. Chinese female players showed no significant differences compared to their global Top 10 counterparts in terms of time-to-success characteristics.

**Conclusion:**

The elite tennis players who achieve success typically start playing and competing in the sport at a young age, with professional competition often commencing around 18 years of age. Notably, these players frequently attain high rankings before reaching the age of 20. Furthermore, top 10 ATP male players tend to start tennis at an earlier age and require a shorter time to achieve a top 10 ranking compared with WTA female players.

## Introduction

To cultivate elite players, a systematic training and development process is essential. By following the law of athletic ability development and implementing properly arranged training phases, maximum effectiveness in the cyclical growth of athletic ability can be achieved [1]. It is important to note that the long-term development characteristics of success differ across sports because of their unique characteristics and physiological basis [2,3]. Therefore, a thorough understanding of the specific developmental characteristics of players is crucial for developing exceptional athletes.

Tennis is a widely popular sport globally, second only to football in terms of its influence. Its competitive and spectator-friendly nature, along with the potential for high prize money, attracts numerous participants and fans. Previous research has delved into the developmental history of tennis players. For instance, Maquirriain’s study [4] found that almost half of the top-ranked female (48.7%) and male (52.5%) players maintained their positions for the following year. Another study, which analyzed 17 years of Grand Slam events, recommended that players commence their professional careers at either the US Open or Wimbledon [5]. Further research has revealed that players who trained on red clay as teenagers have a higher likelihood of reaching the top level in the future [6,7]. This is due to the conditioning effects and longer rallies that result from the greater deformation of tennis on red clay [8,9].

On 24 October 2022, Zhang Zhizhen, a tennis player from China, achieved a historic milestone by being ranked 97th in the new Association of Tennis Professionals (ATP) rankings. This is a significant achievement for China as very few of their players have reached the top 100 in the ATP rankings. It is worth noting that another Chinese player, Wu Yibing, also breaking into the top 100 in the recent rankings and even won China’s first ATP Tour Championship on 23 February 2023. In the women’s events, as many as seven Chinese players have recently entered the top 100 of the Women’s Tennis Association (WTA) rankings. In tennis, the number of players in the top 100 in professional points is considered a crucial indicator of a country’s standard of tennis [10–12], Moreover, most of the tennis Olympic champions are also ranked in the top 30 players in terms of ATP/WTA points. Therefore, the development of more top 100 ATP/WTA players is a critical factor in increasing a country’s impact and competitiveness in tennis.

The ATP/WTA ranking system plays a crucial role in men’s and women’s professional tennis. It tracks a player’s performance over a year and updates their score on a weekly basis. This provides valuable insights into a player’s growth and ability since entering the professional points system [13]. The objective of this study is to investigate the changes in ATP/WTA points of tennis players during professional matches, and to compare the point change patterns of Chinese players with those of elite players, in order to gain a better understanding of the success patterns of top-level tennis players and the potential for future development of Chinese tennis players.

## Materials and methods

### Sample and variable

The data for this study was mainly collected from publicly available sources, including the official ATP website (www.atptour.com), the official WTA website (www.wtatennis.com). In addition, no more than 10% of the data on the age of starting tennis were taken from Wikipedia in conjunction with the results of other web searches. The top 100 ATP and WTA ranked players have been included in the research scope. Furthermore, taking into account the limited number of Chinese athletes in the sample, two relatively high-ranking Chinese players were supplemented as research subjects. A total of 202 players`ranking information was used for analysis. Data acquisition date is October 26, 2022. Since the data for this study were obtained from the internet and no subjects were needed, no ethical proof was required.

### Procedures

Top 600 was used as the starting point for entry into the professional circuit, based on the earlier recommendation of the International Tennis Federation that 600 be considered as seeding for futures events [14]. The moments when the points ranking exceeds 600, 100 and 10 for the first time are used to analyze the growth characteristics of the player. The time taken to enter the top 100 is calculated by subtracting the moment of first entry into the top 600 from the time of first entry into the top 100.

### Statistical analysis

The data were expressed as mean **±** standard deviation, and the data were statistically processed using SPSS22.0 (IBM, Chicago, USA). The K-S method was used to test the normality of the data, the independent samples t-test was used to determine the difference between the two groups of data for normal data, the U-test was used for non-normal data. The Spearman correlation was used to test the correlation of non-normal data, and the Pearson correlation was used to test the correlation of normal data. The criteria for correlation are as follows: 0.1–0.29 = small, 0.3–0.49 = medium, 0.5–0.69 = large, 0.7–0.89 = very large, 0.9–0.99 = almost perfect, and 1 = perfect [15]. Significance level was set at p<0.05.

## Results

**Fig 1:**
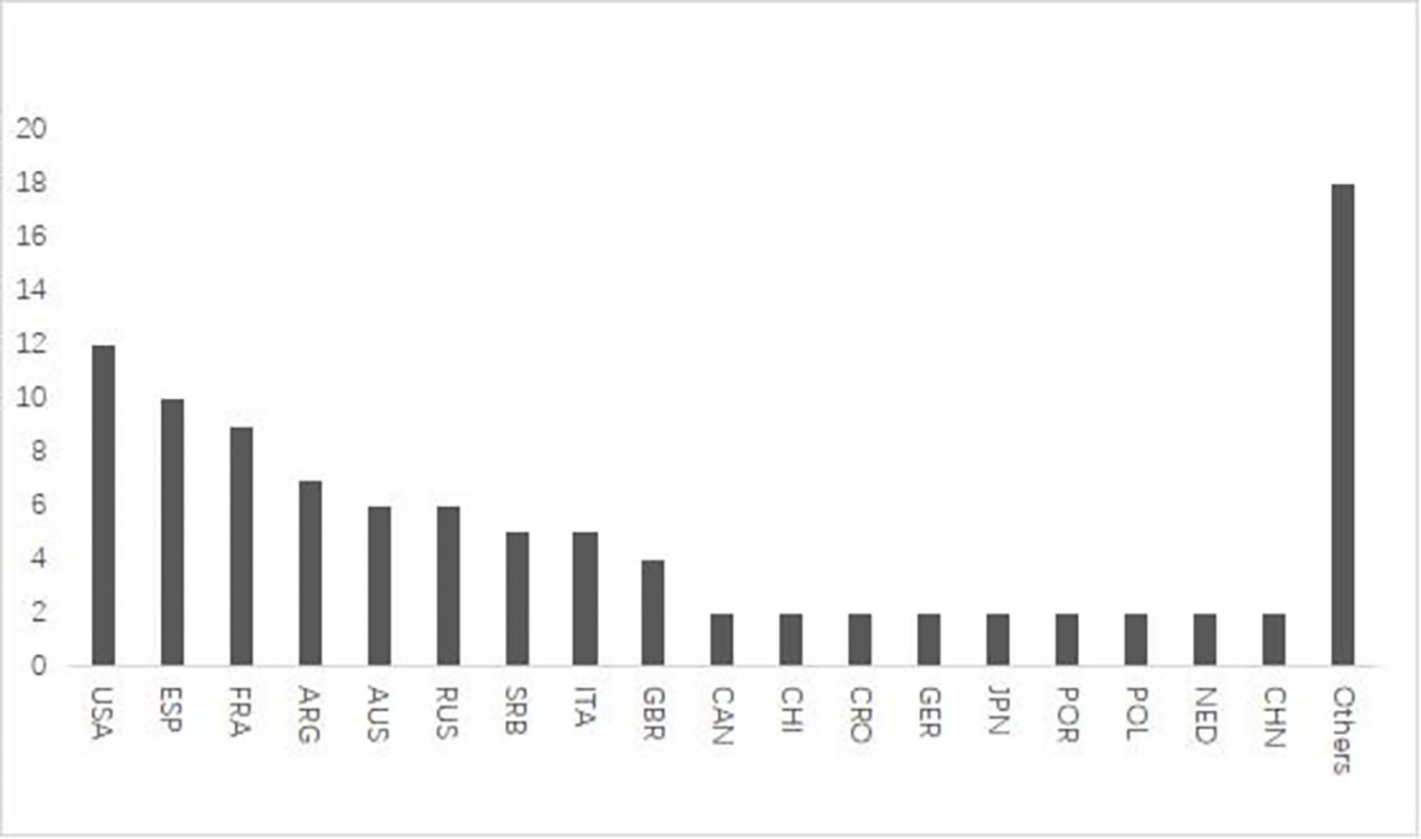
Country distribution of ATP points top 100 players.

**Fig 2:**
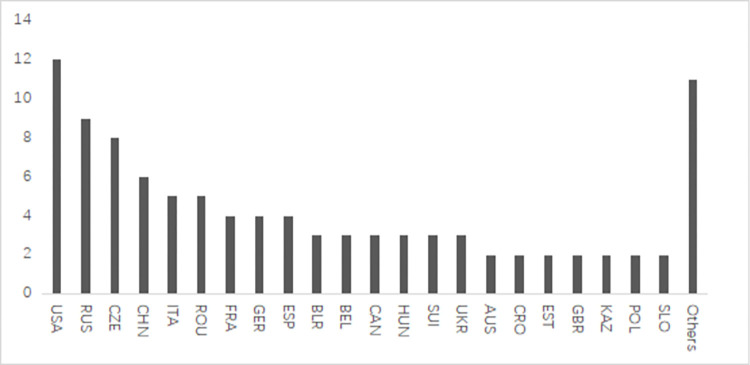
Country distribution of WTA points top 100 players.

To better understand the global competitive landscape of tennis, we counted the nationalities of the top 100 players in the professional standings. Our analysis revealed that 36 countries are represented in the men’s top 100 players (Fig 1), while 33 countries are represented in the women’s top 100 players (Fig 2).

Overall, the majority of top 100 players in professional tennis tournaments are from Europe and the United States, with relatively fewer players hailing from Asia and Africa. The United States leads the world in tennis, with 24 players in the top 100, followed by Russia, Spain and France, with 15, 14, and 13 players respectively, forming the second group. China ranks seventh overall, with a total of eight players in the top 100, with women making up 75% of the overall representation and men comprising 25%, forming the third collective. China has the highest number of top 100 players among all Asian countries, with five more players than Japan and Kazakhstan, which follow as the second-ranked Asian countries.

The average age of the top 100 players in the ATP ranking is approximately 27 years old. These players typically begin tennis at the age of 5, and start ranking at the age of 18. It takes an average of 44 months to enter the top 100, and the average age of entry into the top 100 is around 22 years old. Among Chinese players, Zhang Zhizhen, Wu Yibing, and Shang Juncheng have similar starting ages for tennis and ranking as the overall average, which aligns with the growth characteristics of outstanding players. Additionally, the top 10 players have lower starting ages for tennis, ranking, and entry into the top 100 compared to the average of the top 100 (Table 1).

**Table 1:** World and Chinese elite male players ranking—time characteristics.

	Age	Age start training (years)	Age start ranking (years)	Time to top 100 (months)	Age to top 100(years)
Top 10	25.9±5.43	4.3±1.89	16.5±1.18	21±11.27	18.4±1.17
Top 100	26.72±4.27	5.05±1.80	18.07±1.59	44.17±25.03	21.78±2.94
Zhang Zhizhen (97th)	26	4	18	86	26
Wu Yibing (112th)	23	6	17	/	/
Shang Jun cheng (202nd)	17	5	17	/	/

According to the correlation statistics, the age at which a person begins ranking is significantly positively correlated with their time to reach the top 100, age at which they achieve top 100 status, ranking, and age at which they begin tennis. Time to reach the top 100 is highly positively correlated with the age at which a person achieves top 100 status, and moderately to highly positively correlated with their ranking and age at the start of tennis. The age at which a person achieves top 100 status is also moderately to highly positively correlated with their ranking and age at the start of tennis. However, there is no correlation between ranking and the age at which a person begins tennis (Table 2). To sum up, the above results indicated that the early one player start ranking, the more likely one can reach top 100 early, use less time reach top 100, start tennis early and reach higher ranking; the early one player start tennis, the more likely one can achieve top ranking 100 early; the early one player reach top 100, the higher ranking one may achieve.

**Table 2:** ATP top 100 players ranking—time correlation matrix.

	Age start ranking	Time to top 100	Age to top 100	Ranking
Time to top 100	0.409**			
Age to top 100	0.752**	0.876**		
Ranking	0.287**	0.585**	0.547**	
Age start training	0.318**	0.213*	0.310**	0.143

* represents p<0.05

** represents p<0.01, same below.

Table 3 presents a comparison of the ranking-time characteristics of ATP players ranked in the top 50 versus those ranked 51–100. The findings indicate that the top 50 players had significantly lower ages at the start of their tennis, start of their ranking, time to reach the top 100, and the age at which they achieved top 100 status compared to the 51–100 ranked players (p < 0.05). However, there was no significant difference in age between the two groups (p > 0.05) (Table 3).

**Table 3:** Comparison of ranking time characteristics between ATP top 50 and top 51–100 Athletes.

	Top 50	Top 51–100	T	Z	P
Age	26.34±4.98	27.10±3.43		-1.709	0.087
Age start training(years)	4.76±0.27	5.34±0.23		-2.019	0.044*
Age start ranking(years)	17.70±0.22	18.44±0.21		-2.750	0.006**
Time to top 100(months)	29.28±2.29	59.06±3.32	-29.780		0.000**
Age to top 100	20.12±0.30	23.44±0.39		-5.744	0.000**

The findings reveal that the average age for top female players to commence training worldwide is 5.6 years old, with a starting ranking age of 16.2 years old, and an average age of 23 to enter the world’s top 10. Notably, the starting tennis age and starting ranking age of outstanding players in China are 6.3 years old and 17 years old, respectively, and the difference between them and world-class players is not statistically significant. Similarly, the time taken to reach the 600–100 ranking range is also not statistically significant (Table 4).

**Table 4:** Comparison between Chinese elite female players and the WTA top 10 players.

	Top 10	Chinese elite(n = 7)	T	Z	P
Age start training(years)	5.6±1.36	6.3±2.36	-7.49		0.466
Age start ranking(years)	16.2±0.87	17±0.82	-1.846		0.085
600-400(months)	4.8±6.65	11±11.93		-1.715	0.086
400-200(months)	11.9±7.94	14.29±5.61	-0.388		0.707
200-150(months)	4.9±4.93	7.57±2.98		-0.495	0.621
150-100(months)	17.6±23.94	25.4±22.89		-0.790	0.429

Through the comparative analysis of the top 10 ATP and WTA athletes, it was found that there was no significant difference in the age of starting to rank, the time taken to rank from 600–401, 200–151, 150–101, 100–51, 50–31, 30–11, and the age at which they reached the top 10 between elite male and female athletes. However, there were significant differences in the age of starting tennis, the time taken to rank from 400–201, and the time taken to reach the top 10. Male athletes started tennis at a younger age, took less time to rank from 400–201, and needed a shorter time to reach the top 10 compared to female athletes (Table 5).

**Table 5:** Comparison time characteristics of ATP and WTA top 10 players.

	Male	Female	T	Z	P
Age start training(years)	4.3±1.9	5.6±1.4		-2.076	0.038*
Age start ranking(years)	16.5±1.2	16.2±0.9		-0.364	0.716
600-400(months)	4.6±4.1	4.8±6.6		-0.266	0.790
400-200(months)	5.3±3.4	11.9±7.9	2.241		0.026*
200-150(months)	4.2±3.8	4.9±4.9		-0.267	0.790
150-100(months)	5.8±7.0	17.6±23.9		-1.484	0.138
100-50(months)	8.8±9.0	12.1±9.1		-1.027	0.305
50-30(months)	9.0±8.8	11.4±6.3	0.702		0.492
30-10(months)	13.2±12.0	17±11.2		-0.796	0.426
Age to top 10(years)	20.7±2.1	23±3.4	1.843		0.082
Time to top10(months)	50.9±17.8	79.7±34.5	2.345		0.031*

## Discussion

This study examines the the growth time series characteristics of elite tennis players and compares the disparities between the best Chinese players and their global counterparts. The findings indicate that elite tennis players are mainly from Europe and the Americas, typically begin tennis at the age of 5, commence ranking around 18, and attain a high level of proficiency at around 20. Notably, Chinese female elite players demonstrate no significant differences in their time-related characteristics when compared to top players.

A large improvement in the total number of top 100 players occurred in the United States and China, compared to the data of the previous two decades. An important reason for this phenomenon is the increase in the number of events in these countries. Researchers found that the number of national events has a greater correlation to the competitive level of the nation [16,17]. Since 2004, China has hosted numerous high-level tennis tournaments, such as the China Open, Shanghai Masters, and Wuhan Open. These events have provided domestic players with ample opportunities to compete against world-class players, thereby elevating the overall standard of tennis in China. As a result of participating in these domestic tournaments, players like Zhang Zhizhen, Wu Yibing, and Zheng Qinwen have shown impressive growth, contributing greatly to the country’s success in the sport. Not only in China, but American players have also continued to improve in recent years with the support of a large number of professional tournaments. According to data from October 2001, the United States of America had the second highest number of top 100 players in the world [10], and in 2010 only 12 men and women were in the top 100 in total, while current data shows that their total number of top 100 players has far surpassed the second place. Researchers from the International Tennis Federation show that the correlation between the number of national professional tournaments and the number of players entering the ATP and the number of ATP top 200 players is 0.74 and 0.64 respectively [14], highlight the importance of events for the growth of tennis player. In the context of a large number of professional tournaments, young players are able to gain more opportunities to compete and come into contact with more outstanding players. At the same time, professional tournaments inevitably bring a certain amount of exposure and star effect, which has a significant effect on strengthening the enthusiasm of young tennis participants and enhancing the regional influence of tennis [18]. In fact, the results of this study showed that the age at which the best players entered professional competition showed a high correlation with the age at which they reached the top 100 (r = 0.752), in line with the findings of a previous study by Reid et al [11]. Therefore, authorities should do a better job of hosting professional events and provide more opportunities for players to compete.

However, an athlete’s journey to competition is not begin directly at the professional level, entering a progressive system of competition is extremely important for the mental and physical development of the athlete. In a study by Tennis Australia, the top 20 male and female players on the junior circuit were 51% [6] and 63% [7] more likely to be in the top 100 at the professional level thereafter, and research also showed that the majority of the top 10 players had won at the highest junior level [19]. In addition, retrospective statistics show that the ATP top 250 players play 44–61 matches a year at age 16, while the top 100 players compete in more events at the young level relative to the top 250 [20]. Thus, tennis players who want to achieve in the top level need to participate in enough competitions at the youth level to improve their playing ability.

In addition, our study found that the timing of the start of tennis was extremely important for tennis players and significantly correlated with rankings(p<0.01). Statistics show that the top 10 men’s tennis players start tennis at the age of 4.3 years and women at the age of 5.6 years and enter the professional system around the age of 16, reaching the world’s top 10 in 4 and 6 years for men and women respectively. In a study extending the sample size to the ATP/WTA top 300, it was found that 75% of the players started training at the age of 3–7 [21], further suggesting that tennis is a sport that requires early exposure to training. Our result shows that the timing of participation and peak performance of the best tennis players occurs earlier, with the results of the study showing that the top 50 players start tennis at a relatively earlier age relative to the top 51–100 players in the ATP points system (p<0.05). In addition, study also indicate that the higher the ranking, the earlier the start of the professional system and the earlier the age at which the top 100 was reached, while the top 10 players exited the top 100 until the age of 29 and those ranked 51–100 exited the top 100 at the age of 24.4 [22]. In fact, as ATP/WTA points are calculated cumulatively from the results of previous years of competition, the number one ranking requires a high level of play in all three previous years and a ranking in the top 10 or so in the world three years before the number one ranking is achieved [4], whereas the current ATP number one, Alcaraz, is only 19 years old and previous world champion players such as Nadal achieved the ATP number one before the age of 20. Similarly, the 2012 ATP data shows that players entered the ATP ranking at 16.9 years old and reached the top 100 at 21.5 years old [11]. In 2013, the Australian Tennis Association introduced a version of the expected developmental ranking criteria for top players, which suggested that the top should reach the top 100 at the age of 19 and the top 50 at the age of 20 [13].

The reason that tennis players can train and compete early is that technique is the main factor that determines the level of tennis players. Besides, its key element indicators of fitness are not late maturing qualities such as maximal strength. In fact, tennis spends 75% of its playing time at low intensity levels [23] and average heart rates are in the 60% - 80% maximum heart rate range [24]. A **s**tudy of 86 outstanding young tennis players in the Netherlands also found that maturity and physical fitness levels cannot predict future success [25]. Similarly, research in a large sample size of junior tennis players at national level in Turkey found that tennis playing level was not related to body composition and maturity, but to early training and participation [26]. Despite the controversy that early participation may exacerbate the risk of injury and early retirement, the ITF has introduced appropriate age entry criteria and athlete development programmes, and studies have confirmed the health of junior careers under appropriate protective policies [27,28].

Finally, there is little difference in the growth characteristics of China’s top male and female players compared to other top players, with some players having a greater potential to achieve higher rankings. However, Chinese women athletes usually spend more time in competitions from 600th - 401th place. The reason for this may be associated with the extended adaptation period required for Chinese players entering the professional tournament system, stemming from a lack of mental training and the comparatively delayed development of physical fitness. Study indicate that it is important to train of mental ability for junior tennis players and advocates mental training for youngsters [29]. In the official ITF publication, a study also confirms this point [30]. At the same time, Chinese players face a deficit in scientific physical fitness training during their youth, leading to a disparity between their fitness levels and those of the top players in the initial stages of their careers. And studies also found that fitness levels have a huge correlation to performance for adult players [31,32]. For this reason, the US Tennis Association recommends that 2–3 days of physical fitness training per week be scheduled from the age of 7–8 years old, and advocates that youngsters focus on bilateral imbalances in muscle strength to improve their fitness levels in a coordinated and holistic way [33], which is probably why American tennis standards have improved so rapidly in recent years.

## Conclusions

To summarize, the majority of ATP/WTA top 100 tennis players come from Europe and America, and elite players typically begin tennis at the age of 5, start competing professionally at 18, and achieve a high level of success around the age of 20. In developing tennis players, coaches and practitioners should focus on early training participation, provide more competition opportunities, and emphasize the overall ability of players.

### Limitations

The study presents a few noteworthy limitations. One of these limitations pertains to the absence of training and match details, which hindered our ability to offer specific training and match recommendations for tennis players. Future research should delve into the pathways to success of elite players, offering practitioners a more comprehensive range of information, including factors such as metabolism and diet. Additionally, the age at which tennis training begins, as mentioned in the text, does not conclusively determine the precise pattern. Subsequent research could delve deeper into specific aspects of commencing tennis training, such as the weekly training hours and whether athletes combine it with another sport, to examine its influence on athletes’ long-term development. Furthermore, it’s important to note that correlation analysis does not imply causation, and future longitudinal studies are necessary to ascertain the relationships between various factors and the long-term development of tennis players.

## Supporting information

S1 File Raw data. (DOC)
