# Supplementary material for: Development of youth tennis players: A study based on the ranking history of top ATP/WTA players worldwide and China
Source: PLoS One. 2023 Nov 10;18(11):e0289848. doi: 10.1371/journal.pone.0289848 (PMC10637680; doi:10.1371/journal.pone.0289848)
Supplement: S1 File — (DOC) [file pone.0289848.s001.doc]

S1 File. The raw data of the development of youth tennis players: a study based on the ranking history of top ATP/WTA players worldwide and China.

https://figshare.com/articles/dataset/Development_of_Youth_Tennis_Players_A_Study_Based_on_the_Ranking_History_of_Top_ATP_WTA_Players_Worldwide_and_China/24187413
